# Synthetic DNA fragments bearing ICR *cis* elements become differentially methylated and recapitulate genomic imprinting in transgenic mice

**DOI:** 10.1186/s13072-018-0207-z

**Published:** 2018-06-29

**Authors:** Hitomi Matsuzaki, Eiichi Okamura, Daichi Kuramochi, Aki Ushiki, Katsuhiko Hirakawa, Akiyoshi Fukamizu, Keiji Tanimoto

**Affiliations:** 1 Faculty of Life and Environmental Sciences, University of Tsukuba, Tennoudai 1-1-1, Tsukuba, Ibaraki 305-8577 Japan; 2 Life Science Center for Survival Dynamics, Tsukuba Advanced Research Alliance (TARA), University of Tsukuba, Tsukuba, Ibaraki 305-8577 Japan; 3 Graduate School of Biomedical Sciences, Tokushima University, Tokushima, 770-8503 Japan; 4 Graduate school of Life and Environmental Sciences, University of Tsukuba, Tsukuba, Ibaraki 305-8577 Japan

**Keywords:** Genomic imprinting, DNA methylation, *H19*, CTCF, Sox-Oct, ZFP57

## Abstract

**Background:**

Genomic imprinting is governed by allele-specific DNA methylation at imprinting control regions (ICRs), and the mechanism controlling its differential methylation establishment during gametogenesis has been a subject of intensive research interest. However, recent studies have reported that gamete methylation is not restricted at the ICRs, thus highlighting the significance of ICR methylation maintenance during the preimplantation period where genome-wide epigenetic reprogramming takes place. Using transgenic mice (TgM), we previously demonstrated that the *H19* ICR possesses autonomous activity to acquire paternal-allele-specific DNA methylation after fertilization. Furthermore, this activity is indispensable for the maintenance of imprinted methylation at the endogenous *H19* ICR during the preimplantation period. In addition, we showed that a specific 5′ fragment of the *H19* ICR is required for its paternal methylation after fertilization, while CTCF and Sox-Oct motifs are essential for its maternal protection from undesirable methylation after implantation.

**Results:**

To ask whether specific *cis* elements are *sufficient* to reconstitute imprinted methylation status, we employed a TgM co-placement strategy for facilitating detection of postfertilization methylation activity and precise comparison of test sequences. Bacteriophage lambda DNA becomes highly methylated regardless of its parental origin and thus can be used as a neutral sequence bearing no inclination for differential DNA methylation. We previously showed that insertion of only CTCF and Sox-Oct binding motifs from the *H19* ICR into a lambda DNA (LCb) decreased its methylation level after both paternal and maternal transmission. We therefore appended a 478-bp 5′ sequence from the *H19* ICR into the LCb fragment and found that it acquired paternal-allele-specific methylation, the dynamics of which was identical to that of the *H19* ICR, in TgM. Crucially, transgene expression also became imprinted. Although there are potential binding sites for ZFP57 (a candidate protein thought to control the methylation imprint) in the larger *H19* ICR, they are not found in the 478-bp fragment, rendering the role of ZFP57 in postfertilization *H19* ICR methylation a still open question.

**Conclusions:**

Our results demonstrate that a differentially methylated region can be reconstituted by combining the activities of specific imprinting elements and that these elements together determine the activity of a genomically imprinted region in vivo.

**Electronic supplementary material:**

The online version of this article (10.1186/s13072-018-0207-z) contains supplementary material, which is available to authorized users.

## Background

A small subset of autosomal genes in mammals is expressed only from one parental allele because of genomic imprinting. This mono-allelic gene expression pattern is essential for normal development, and its failure results in human diseases including Beckwith–Wiedemann and Silver–Russell syndromes [1, 2]. The imprinted genes are marked by epigenetic modifications, among which allele-specific DNA methylation at the imprinting control regions (ICRs) plays a pivotal role in their unique expression pattern, as demonstrated in DNA methyltransferase deficient mice [3–5] and ICR-knockout mice [6–8].

Because differential methylation of the ICRs is acquired during either oogenesis or spermatogenesis, these sequences are also called the germline differentially methylated regions (gDMRs). It has long been predicted that there is a specific mechanism by which the ICRs are specifically targeted for de novo methylation in germ cells [9, 10]. Recent studies, however, revealed that genomic regions other than the ICRs are also methylated during gametogenesis, suggesting that gametic methylation at the ICRs occurs as only part of a broad de novo methylation program [11–13]. After fertilization, however, while most gamete-derived methylation is lost, allelic methylation at the ICRs is faithfully retained to control imprinted gene expression thereafter. We therefore assume that a specific mechanism, by which allelic methylation is maintained at restricted sequences against genome-wide epigenetic reprogramming during preimplantation development, defines imprinted genomic loci [14].

**Fig. 1:**
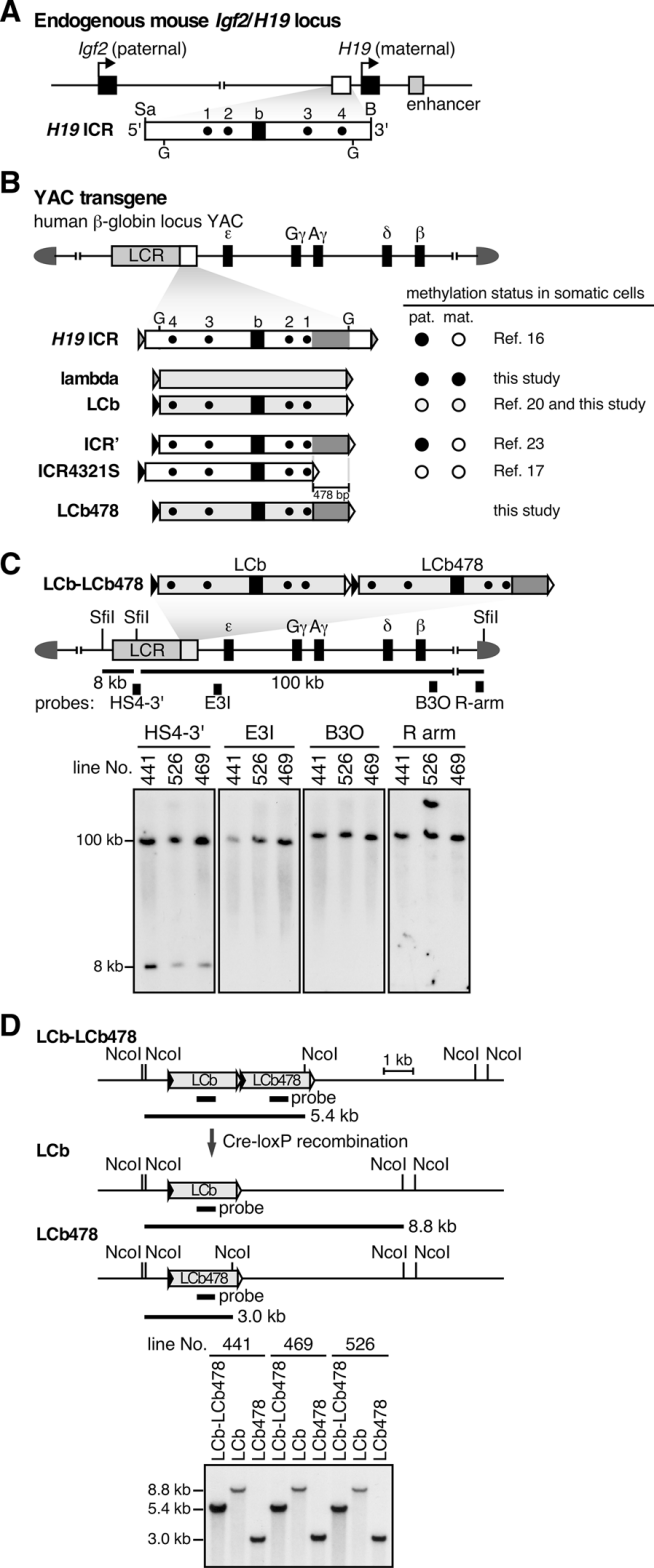
Experimental design. **A** Structure of the mouse *Igf2/H19* locus. The expression of paternal *Igf2* and maternal *H19* genes depends on the shared 3′ enhancer. The *H19* ICR, located approximately at − 4 to − 2 kb relative to the transcription start site of *H19* gene is contained within a 2.9-kb *Sac*I (Sa)-*Bam*HI (B) fragment. Dots (1–4) and a filled box in *H19* ICR indicate CTCF-binding sites and the “b” region, respectively. G; *Bgl*II site. **B** Structure of the 150-kb human β-globin locus YAC. The LCR and β-like globin genes are denoted as gray and filled boxes, respectively. The *H19* ICR (2.9-kb *H19* ICR, 2.4-kb ICR’ and ICR4321S) or lambda (lambda, LCb and LCb478) fragments were introduced 3′ to the LCR. Their methylation states after paternal (pat.) or maternal (mat.) transmission determined in our previous studies [16, 17, 20, 23] are summarized on the right. YAC–TgM carrying the LCb/LCb478 fragments were generated in this study. The different pairs of loxP sites (loxP [gray]/loxP5171 [solid]/loxP2272 [open]) are shown as triangles. **C** Long-range structural analysis of the LCb–LCb478 YAC transgene. The expected *Sfi*I restriction enzyme fragments (thick lines) and probes (filled rectangles) are shown. The enlarged map shows tandemly arrayed LCb and LCb478 fragments, inserted 3′ to the LCR for employing co-placement strategy [24]. DNA from thymus cells was digested with *Sfi*I in agarose plugs and separated by pulsed-field gel electrophoresis, and Southern blots were hybridized separately to probes. **D** In vivo Cre-loxP recombination to derive LCb or LCb478 TgM. Recombination between two loxP5171 sites (solid) in the parental LCb–LCb478 transgene, for example, would generate LCb478 allele, during which one of the loxP2272 sites (open) is concomitantly removed to prevent further recombination. Tail DNA from parental and daughter YAC–TgM sublines was digested with *Nco*I and analyzed by Southern blotting using the probe

Our recent studies on the *H19* ICR of the *Igf2/H19* gene locus support the existence of such a preimplantation methylation maintenance mechanism. The *H19* ICR, located on mouse chromosome 7 and human chromosome 11, controls preferential expression of the *Igf2* and *H19* genes on the paternal and maternal alleles, respectively (Fig. 1A). Once methylated in pro-spermatogonia, the ICR status is maintained on the paternal allele beyond fertilization [15]. We tested its autonomy in yeast artificial chromosome (YAC) transgenic mice (TgM), in which a mouse *H19* ICR fragment (2.9 kb) was inserted into a YAC bearing the non-imprinted human β-globin locus (150 kb, Fig. 1B, [16]). Although the transgenic *H19* ICR sequence was not methylated in sperm, it was preferentially methylated in offspring only after paternal transmission. This allele-specific DNA methylation, which commenced soon after fertilization, required the oocyte-derived de novo methyltransferases, *Dnmt3a* and *Dnmt3L* [17]. These results demonstrated that the *H19* ICR sequence possesses an intrinsic activity allowing it to acquire allele-specific DNA methylation after fertilization. In addition, when methylation of the endogenous *H19* ICR was experimentally obstructed in male germ cells, it was restored after fertilization by the action of de novo methyltransferases [17], demonstrating that allele-specific, postfertilization methylation also takes place at the endogenous locus. We thus proposed that this de novo methylation activity contributed to the maintenance of paternal methylation at the *H19* ICR during preimplantation development. Importantly, a 5′-truncated *H19* ICR fragment, which was 765-bp shorter than the tested 2.9-kb sequence, failed to acquire methylation after fertilization both at endogenous, as well as in transgenic loci, although its methylation status in sperm was unchanged [17]. It therefore seemed most likely that specific sequences within the 5′-segment of the *H19* ICR are involved in the postfertilization imprinted methylation mechanism.

In contrast, we and others have found that during the postimplantation period, the protection from de novo methylation of a maternal unmethylated *H19* ICR was essential for maintenance of its differentially methylated state. To date, two *cis* elements, CTCF-binding sites and Sox-Oct motifs within the *H19* ICR, have been shown to be essential for this process, since mutation of these elements causes aberrant methylation of the maternal ICR after implantation [18–21].

These results collectively suggested that the differentially methylated state of the *H19* ICR is governed by distinct processes during gametogenesis, preimplantation, and postimplantation, but among which the mechanisms after fertilization are more decisive in determination of imprinting. Furthermore, in contrast to the gametic methylation process, in which widespread transcriptional or histone modification states appear to be involved [13, 14], postfertilization differential methylation of the *H19* ICR is predicted to be controlled by the combinatorial action of specific *cis* regulatory elements within the ICR. In this study, we demonstrate that a reconstituted DNA fragment, composed of multiple *cis* regulatory sequences found in the *H19* ICR, are capable of recapitulating appropriate imprinted methylation dynamics after fertilization.

## Results

### Generation of YAC–TgM carrying a reconstituted fragment

**Fig. 2:**
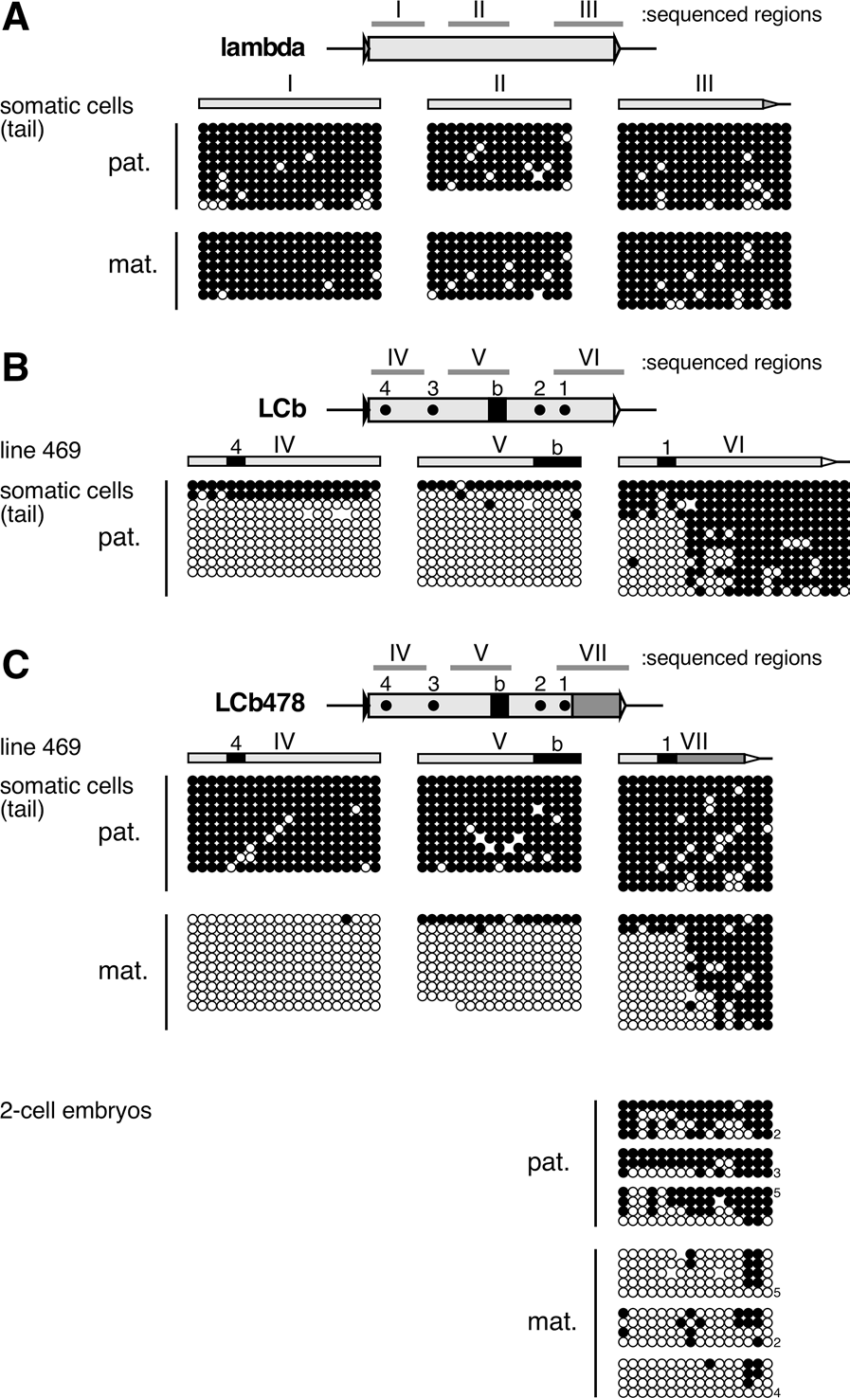
The LCb478, but not LCb, fragments acquire paternal-allele-specific methylation in YAC–TgM. Tail somatic DNA from the lambda (**A**), LCb (**B**) or LCb478 (**C**), and DNA from 2-cell embryos of the LCb478 (**C**) YAC–TgM, inheriting the transgenes either paternally (pat.) or maternally (mat.), were analyzed by bisulfite sequencing. Methylated and unmethylated CpG motifs are shown as filled and open circles, respectively, and each horizontal row represents a single DNA template molecule. The results of 2-cell-embryo DNA from a single litter are presented together in a cluster. The numbers on the right of each row indicate number of times the pattern was observed by DNA sequencing

Using mouse genetic approaches, we have dissected *H19* ICR activity and identified multiple *cis* elements essential for protecting the sequence from undesirable methylation of the maternal allele, as well as one capable of conferring methylation to the paternal allele, both after fertilization. To test whether these elements are *sufficient* to generate imprinted methylation status, we conducted presumptive reconstitution experiments. We started by employing a 2.3-kb bacteriophage lambda DNA fragment as a “neutral” sequence, as it resembles the *H19* ICR in both size and CpG frequency (Fig. 1B). When it was inserted into a β-globin YAC (Fig. 1B, [22]), the fragment became highly methylated regardless of its parental origin in YAC–TgM (Fig. 2A). Because CTCF-binding sites and Sox-Oct binding motifs are required to maintain the unmethylated state of the maternal *H19* ICR [18, 20], we transplanted these elements [four CTCF sites and two copies of Sox-Oct motifs (“b” sequence) were introduced at the same time] into the lambda DNA in an arrangement that was comparable to that found in the natural *H19* ICR, and designated the fragment “LCb” (Lambda with CTCF and b sequences) (Fig. 1B, [20]). We anticipated that this combination of *cis* elements would prevent methylation only after maternal transmission. In YAC–TgM, however, the LCb fragment became hypo-methylated after either paternal or maternal transmission, demonstrating that CTCF and b sequences together conferred non-selective activity to both parental alleles in establishing their unmethylated DNA status.

This result prompted us to attempt to identify the sequence that must be capable of introducing paternal allele-specific, postfertilization DNA methylation into LCb. We previously found that the “ICR’” sequence, which was originally identified as a 2.4-kb fragment by *Bgl*II digestion of mouse genomic DNA, was differentially methylated in YAC–TgM (Fig. 1B, [23]), while a smaller (ICR4321S) fragment, missing 478-bp from the 5′ end of ICR’, failed to acquire methylation in YAC–TgM even after paternal transmission (Fig. 1B, [17]). The importance of that 5′-segment of the *H19* ICR was also demonstrated by mutagenesis of the endogenous locus: When a 765-bp fragment that included the 478-bp sequence was deleted, the *H19* ICR lost its ability to methylate the paternal allele after fertilization [17]. These results suggested that the 478-bp sequence at the 5′-end of the *H19*-ICR contained the sought after allele-specific, postfertilization methylation-inducing activity. We therefore added this sequence to LCb and annotated it as “LCb478” (Fig. 1B).

We asked whether LCb478 could acquire allele-specific differential DNA methylation in YAC–TgM when compared to LCb. To precisely compare the activities of these two fragments when integrated at the identical genomic location, the LCb and LCb478 fragments were individually floxed using two distinct pairs of loxP sequences (loxP5171 or loxP2272), and then both fragments were inserted in tandem to enable a transgene co-placement strategy (Fig. 1C, [24]). The floxed fragments were inserted into a human β-globin YAC at a position 3′ to the locus control region (LCR), and three TgM lines were established. The copy number and long-range structural analyses (Fig. 1C) of these mice showed that lines 441 and 469 carried a single, intact copy of the integrated YAC transgene, while line 526 carried a single, intact YAC copy plus a right arm fragment. Each parental YAC line was crossed with Cre-TgM to promote in vivo Cre-loxP recombination, which generated daughter lines carrying either the LCb or LCb478 transgene at the identical chromosomal integration site (Fig. 1D).

### Postfertilization imprinted methylation is recapitulated by LCb478

We examined the methylation status of transgenes in the somatic cells of the YAC–TgM. When analyzed by Southern blotting using a methylation-sensitive restriction enzyme, the control LCb fragments became hypo-methylated regardless of their parental origin, although the penetrance seemed incomplete after paternal transmission (Additional file 1: Fig. S1A and B). Bisulfite sequencing of the pooled samples revealed that the methylation levels of the paternal LCb allele were quite low, especially in regions IV and V (Fig. 2B). Although the CpGs outside of CTCF site 1 in region VI was substantially methylated, this methylation seemed to be acquired independently of its parental origin (i.e., non-DMR; see below and [20]). These results confirmed our previous conclusion that the LCb sequence was not sufficient to generate a differentially methylated state [20].

**Fig. 3:**
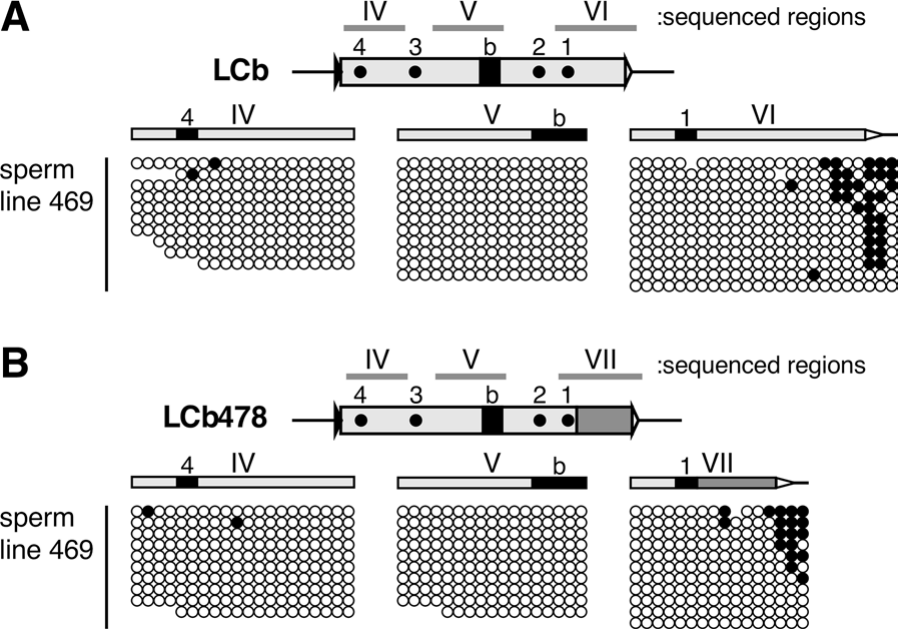
LCb and LCb478 fragments are unmethylated in sperm. Sperm genomic DNA from adult male TgM was analyzed by bisulfite sequencing as described in the legend to Fig. 2

In contrast, when integrated at the identical chromosomal position, the LCb478 became highly methylated only when it was paternally inherited in all three TgM lines (Additional file 1: Fig. S1C and D), as confirmed (in regions IV and V) by bisulfite sequencing (Fig. 2C; somatic cells). Because the CpG motifs located outside of CTCF site 1 became highly methylated after both paternal and maternal transmission, the border between DMR and non-DMR sequences seemed to be established within region VII. Importantly, the imprinted methylation of fragment LCb478 was detected in as early as 2-cell stage embryos (Fig. 2C; 2-cell embryos). Because both the LCb478 and LCb fragments were not methylated in testes (Additional file 2: Fig. S2) and sperm (Fig. 3), it is apparent that paternal-allele-specific methylation of LCb478 was acquired only after fertilization. These results demonstrate that the postfertilization imprinted methylation observed in the *H19* ICR transgene was completely recapitulated by LCb478, which was reconstituted by combining the activities of distinct regulatory elements identified in the *H19* ICR.

### Imprinted methylation of the LCb478 is likely to be acquired by a ZFP57-independent mechanism

**Fig. 4:**
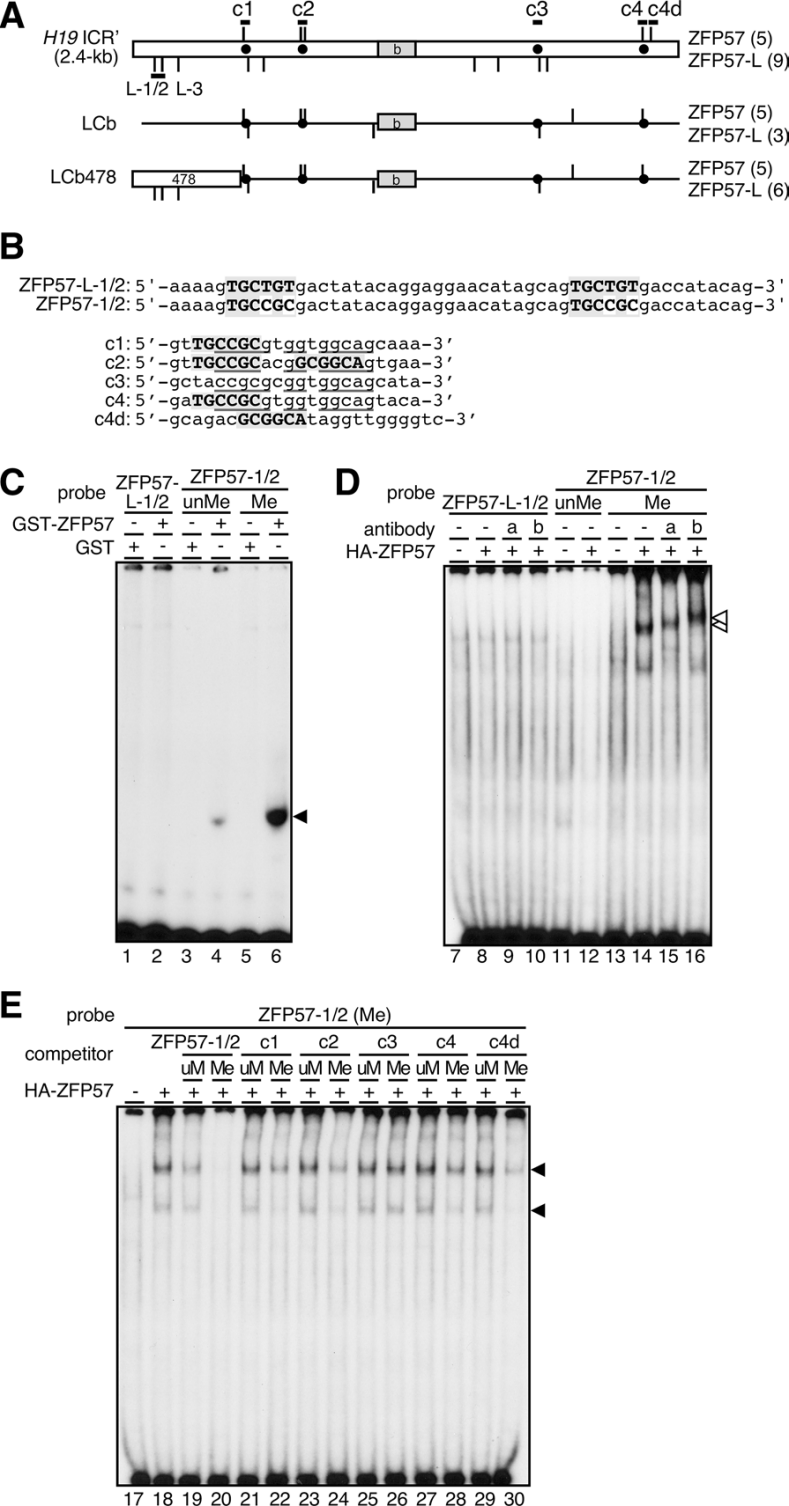
Analysis of ZFP57 binding to the LCb478 sequence in EMSA. **A** Distribution of ZFP57 (above the line) and ZFP57-like (L; below the line) motifs in the *H19* ICR’ (top), LCb (middle) and LCb478 (bottom) sequences. CTCF-binding sites and b sequence are indicated by black circles and a gray rectangle, respectively. Position of probes and competitors used for EMSA in panels **C**–**E** is shown as thick horizontal lines. **B** (top) Two out of six nucleotides in the consensus ZFP57 binding site-like sequences (shaded) in the ZFP57-L-1/2 probe were mutated to generate canonical ZFP57 binding sites in the ZFP57-1/2 probe. (bottom) Sequence of competitor oligos each carrying CTCF-binding sites (c1-c4 or c4d [downstream]) within the *H19* ICR (yet outside of the 478 sequence). Consensus CTCF and putative ZFP57 binding sites are underlined or shaded, respectively. **C** GST or GST-ZFP57 (a.a. 137–195) fusion proteins were expressed in and recovered from *E. coli*. The GST-ZFP57 protein bound robustly to the methylated ZFP57-1/2 probe (lane 6), but not to its unmethylated counterpart (lane 4) or to the ZFP57-L-1/2 probe (lane 2). **D** Nuclear extracts of HEK293T cells transfected with (+) or without (−) a HA-ZFP57 expression vector were analyzed by ZFP57-L-1/2 or ZFP57-1/2 probes. Combination of cell extracts with forcibly expressed HA-ZFP57 and methylated ZFP57-1/2 probe generated shifted bands (lane 14). The antibody/HA-ZFP57 super-shifted bands are denoted by the open arrowheads (lanes 15 and 16). antibodies a: α HA; b: α ZFP57. **E** Competitor oligos (50-fold molar excess) were included in the EMSA with HEK293T/HA-ZFP57 nuclear extracts and methylated ZFP57-1/2 probe, to test ZFP57 binding to the LCb/LCb478 sequences

It has been reported that TRIM28/TIF1β/KAP1 protein was required for the maintenance of methylation of several ICRs, including the *H19* ICR, in preimplantation embryos [25]. ZFP57, a member of the KRAB-Zn finger protein families, preferentially binds methylated DNA sequences and can mediate the interactions between TRIM28 and imprinted loci [26]. Because ZFP57 protein interacts not only with Dnmt1 maintenance methyltransferase, but also with Dnmt3 de novo methyltransferases [26, 27], we postulated that this transcription factor might be involved in postfertilization methylation acquisition. There are five consensus binding motifs for ZFP57 within the *H19* ICR’; four of them overlap with CTCF sites 1 (c1), 2 (c2), and 4 (c4), and another is located downstream of CTCF site 4 (c4d) (Fig. 4A, B). When we generated the LCb fragment, the first four were fortuitously transplanted into the lambda “null” DNA, accompanying the CTCF-binding sequence insertion (Fig. 4A). Nonetheless, this fragment failed to acquire paternal-allele-specific methylation (Additional file 1: Figs. S1B, 2B, [20]), indicating that these canonical ZFP57 motifs were not sufficient to establish postfertilization imprinted methylation in the LCb sequence.

We discovered three DNA sequences those were similar but not identical to the consensus ZFP57 binding motif [26, 28] in the 478-bp region (Fig. 4A, B). We therefore named the motif “ZFP57-L (-like)”, and tested their binding potential to ZFP57 by EMSA, to ask whether they might contribute to methylation acquisition. We employed 51-bp (ZFP57-L-1/2) or 20-bp (ZFP57-L-3) double-stranded DNA fragments as probes, each bearing two or one copy of the ZFP57-L motif, respectively (Fig. 4A, B and not shown). However, recombinant ZFP57 protein (amino acids 137–195; GST-ZFP57 [29]) expressed in *Escherichia coli* did not bind to the probes (Fig. 4C and not shown). We then converted the ZFP57-L motif to the one that was identical to the canonical ZFP57 binding motif in the ZFP57-L-1/2 probe (Fig. 4B; ZFP57-1/2). When the latter was methylated in vitro and tested by EMSA, ZFP57 binding to the probe was clearly detectable (Fig. 4C). The binding ability of the probes was also tested using forcibly expressed ZFP57 protein in the HEK293T cell nuclear extract (Fig. 4D). Again, the protein bound exclusively to the in vitro methylated ZFP57-1/2 probe, the specificity of which was confirmed in a super-shift assay (Fig. 4D). We also determined that methylated DNA fragments containing ZFP57-binding motifs that are present near the CTCF sites weakly yet significantly interacted with ZFP57 (as evidenced by competition experiments; Fig. 4D; competitor: c1, c2, c4 and c4d), even though the LCb did not acquire paternal methylation. We tentatively conclude that postfertilization imprinted methylation at the *H19* ICR requires regulation by factor(s) other than ZFP57 protein binding to the 478-bp sequence.

### The LCb478 fragment regulates imprinted expression in a YAC transgene

**Fig. 5:**
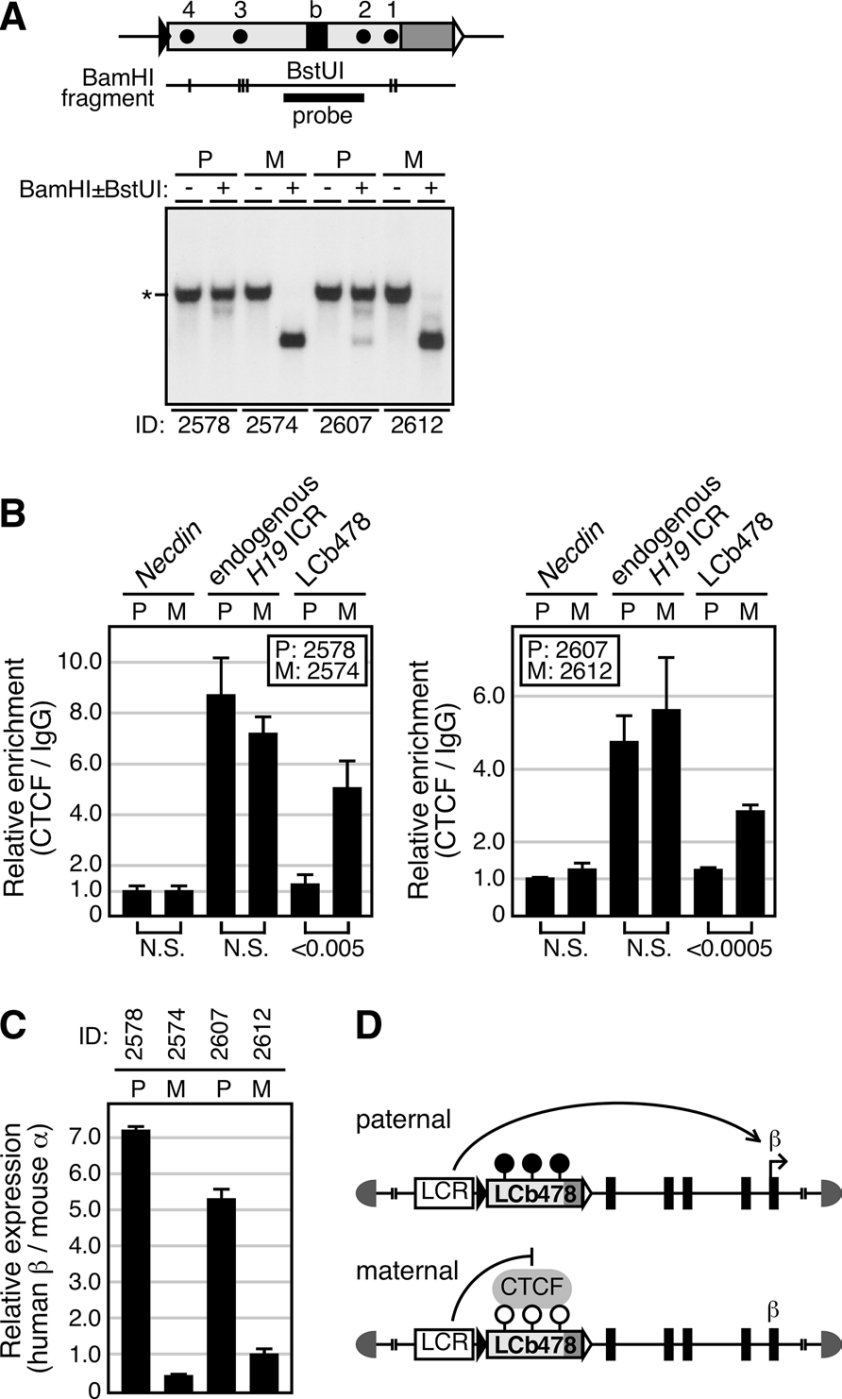
Genomic imprinting is recapitulated in LCb478 YAC-TgM. **A**–**C** Two pairs of LCb478-TgM (2578 vs 2574 and 2607 vs 2612, 1 month old), each inheriting the transgene either paternally (P) or maternally (M) were made anemic and spleens were removed, from which one-quarter each was used for genomic DNA or total RNA preparation with the remaining half used for chromatin preparation. **A** DNA methylation status of the transgene was determined by Southern blot analysis using *Bam*HI with (+) or without (−) *Bst*UI (vertical lines) and a probe. *: parental or methylated, uncut fragments. **B** ChIP analysis of CTCF occupancy at the transgene. Chromatin was immunoprecipitated using either control IgG or anti-CTCF antibodies. Following qPCR analyses of three distinct genomic regions (*Necdin*; negative control, endogenous *H19* ICR; positive control, and LCb478), relative enrichment values (CTCF/IgG signal ratio) were calculated. The average and standard deviation (S. D.), determined by three reactions, are depicted, as a signal for *Necdin* (P) was arbitrary set at 1.0. **C** The relative expression levels of the human β-globin gene, after normalization to that of the endogenous mouse α-globin gene were determined by RT-qPCR analysis. The average and standard deviation (S.D.), determined by three reactions, are depicted, as a value of No. 2612 animal was arbitrary set at 1.0. **D** Schematic representation of the genomic imprinting recapitulated in the transgene

We next examined whether or not the differentially methylated LCb478 could confer imprinted regulation of gene transcription. At the *Igf2/H19* locus, the maternal allele-specific insulator activity is governed by CTCF-binding to the unmethylated *H19* ICR and prevents activation of the *Igf2* gene by a 3′-downstream enhancer, resulting in its expression exclusively from the paternal allele. In YAC–TgM, the LCb478 was methylated only after paternal transmission in erythroid cells (Fig. 5A). ChIP assays revealed that CTCF was enriched two to three times on the maternal, unmethylated LCb478 allele than on the paternal, methylated sequence, where enrichment was as little as that seen in the negative control (*Necdin*) locus (Fig. 5B). Furthermore, transgenic β-globin gene expression was significantly suppressed after maternal transmission (Fig. 5C), suggesting that CTCF-dependent insulator activity formed by the maternal LCb478 sequences prevented β-globin gene activation by the LCR “superenhancer” (Fig. 5D). Taken together, these results demonstrated that the reconstituted LCb478 fragment was not only able to confer allele-specific methylation to CpG residues in a parent of origin dependent manner, but was also able to control imprinted gene expression.

## Discussion

Currently, it is generally accepted that de novo DNA methylation of the ICRs in germ cells is under the control of transcription that traverses their sequence and through their association with unmethylated histone H3 lysine-4 signatures [13, 14]. Thus compelling evidence has shown that distinct chromatin states in male and female germlines define different methylation levels at the ICRs. While non-ICR genomic DNA sequences with the same chromatin features, however, are also methylated during gametogenesis, most of these gamete-derived non-ICR DNA methylations are lost during preimplantation development after fertilization [11, 12]. Therefore, allele-specific activities for preventing the loss of DNA methylation at specific genomic sites must exist in preimplantation embryos to faithfully maintain the differential methylation status of the ICRs.

Postfertilization maintenance of differential methylation of the ICRs can be divided into two distinct stages: One is protection of the methylated ICRs from genome-wide DNA *de*methylation as part of epigenetic reprograming in preimplantation embryos, and the other is protection of an unmethylated ICR against undesirable de novo methylation after implantation. At the *H19* ICR, we previously proposed that postfertilization, allele-specific de novo DNA methylation was responsible for the former event [17]. Our current results clearly demonstrated that 478-bp corresponding to the 5′-portion of the *H19* ICR is sufficient to instruct paternal-allele-specific methylation during preimplantation development (Fig. 2C).

Since TRIM28 has been reported to be necessary for methylation maintenance at multiple ICRs, including the *H19* ICR during this period [25], the 478-bp sequence may be the platform for this factor to contribute de novo methylation of the *H19* ICR. TRIM28, however, has no intrinsic DNA-binding ability and thus requires association with other sequence-specific DNA-binding protein(s) for recruitment to a target site. Although ZFP57, which is capable of binding to TRIM28, was a preferred candidate for such a factor [26], we could not demonstrate its interaction with consensus motif-like sequences within the 478-bp region (Fig. 4), suggesting that ZFP57 is not required for the function of the 478-bp sequence. In addition, this result is consistent with reports showing that the methylation level of the *H19* ICR is not affected in *Zfp57* knock-out mice [30, 31].

Previously, we and others have shown that CTCF-binding sites [18, 19] and Sox-Oct motifs [20, 21] are both necessary to prevent undesirable methylation in the maternal *H19* ICR during the postimplantation period. On the one hand, at the maternally methylated ICRs [32], protection against undesired methylation activity in the paternal allele has been suggested to be important for differential methylation maintenance. One might therefore presume that active involvement of maintenance mechanisms for both methylated and unmethylated alleles is essential for the generation of a differential methylation state on both the paternally and maternally methylated ICRs.

It will be intriguing to examine whether both paternal and maternal allele-specific mechanisms are also operative at other ICRs, and whether or not the regulatory elements in the *H19* ICR are shared with them. For example, whether CTCF-binding sites in the ICRs of the *Rasgrf1*, *Kcnq1/Kcnq1ot1*, and *Grb10* loci [33–35] are also required for protecting them from genome-wide methylation after implantation is yet to be determined. In addition, it was recently reported that histone H3-lys27 methylation, instead of DNA methylation, was used as chromatin signature to discriminate parental origin of the ICRs at some imprinted loci [36]. We found that *H19* ICR could acquire allele- and region-specific DNA methylation even after fertilization independently from its gametic methylation status [16]. Therefore, it will be of primary significance to reveal whether such histone modifications are set within the *H19* ICR during gametogenesis and used to distinguish its parental origin for imprinted methylation during the postfertilization period, and if their states are under the control of the 478-bp sequence.

## Conclusions

We demonstrated that postfertilization imprinted methylation, as well as its related imprinted gene expression pattern, can be fully reconstituted by combining specific *cis* elements in mice; to our knowledge this comprises the very first example of genomic imprinting recapitulation by synthetic elements. Because loss of imprinting causes abnormal development and a variety of human diseases, understanding the underlying molecular mechanisms is paramount. Our findings here restrict the range of candidate *cis* elements as potential therapeutic targets and provide useful tools to investigate their roles in the pathogenesis of imprinting diseases.

## Methods

### Generation of the LCb478 fragment

The 5′-end portion of the LCb478 fragment was PCR-generated using the murine *H19* ICR DNA as a template and a set of primers: 5′del_fr-3A9+B, 5′-GAAGAGATCTGGATCCAGCTCTATCCCATCGAAA-3′ (*Bgl*II and *Bam*HI sites are underlined) and MluI-CTCF1-lambda-3A, 5′-TCCGCACGCGTTTTG*CTGCCACCACGCGG*CAACtaggtgtttTAAACCCCACAACTGATTCA-3′ (*Mlu*I and CTCF-binding sites underlined and italicized, respectively; *λ* sequences are shown in lower case letters). The resultant fragment was digested with *Bgl*II/*Mlu*I and used for following construction steps.

Preparation of *λ* + CTCF + b (LCb) sequences were described elsewhere [20]. The LCb fragment, released by *Bam*HI digestion was blunt-ended and ligated with *Bgl*II linker (pCAGATCTG). The 3′-segment of this fragment, carrying CTCF sites 2–4, was recovered by *Mlu*I/*Bgl*II digestion and linked to 5′-end portion of the LCb478 (*Bgl*II-*Mlu*I fragments, described above) to generate the LCb478 fragment.

### Yeast targeting vectors and homologous recombination in yeast

The co-placement target vector, pHS1/loxP-5171-B-2272-5171-G-2272 (pCop5B25G2), in which 5′-loxP5171-*Bam*HI-loxP2272-loxP5171-*Bgl*II-loxP2272-3′ sequences are introduced at *Hin*dIII site [at nucleotide 13,769 (HUMHBB; GenBank)] of the human β-globin HS1 fragment [nucleotides 13,299–14,250 in HUMHBB], was described elsewhere [20].

The LCb fragment was inserted into *Bam*HI site of pCop5B25G2 to generate pCop5[LCb]25G2. The resultant plasmid was digested with *Bgl*II and ligated with another fragment, the LCb478 to generate pCop5[LCb]25[LCb478]2 (Fig. 1C). In each cloning step, the correctness of DNA construction was confirmed by DNA sequencing.

The targeting vector was linearized with *Spe*I [at nucleotide 13,670 in HUMHBB] and used to mutagenize the human β-globin YAC (A201F4.3) [37]. Successful homologous recombination in yeast was confirmed by Southern blot analyses with several combinations of restriction enzymes and probes.

### Generation of YAC–TgM

Purified YAC DNA was microinjected into fertilized mouse eggs from C57BL/6J (Charles River) mice. Tail DNA from founder offspring was screened first by PCR, followed by Southern blotting. Structural analysis of the YAC transgene was performed as described elsewhere [37, 38]. The Zp3-Cre TgM (Jackson Laboratory) [39] was mated with parental YAC–TgM lines to derive sublines carrying either LCb or LCb478 sequences (co-placement strategy, [24]). Successful Cre-loxP recombination was confirmed by Southern blotting (Fig. 1D).

TgM carrying a human β-globin YAC, in which the lambda fragment was inserted between the LCR and the ε-globin gene (Fig. 1B) were described previously (“HS1/*λ*”) [22].

### Preparation of embryos

Female mice were super-ovulated via injection of pregnant mare serum gonadotropin, followed by human chorionic gonadotropin (hCG) (47–48-h interval). Two-cell embryos were flushed from oviducts by M2 medium at 44 h after hCG injection, and then washed by PBS.

### DNA methylation analyses

For Southern blot analysis, genomic DNA from tail tips of ~ 1-week-old animals, adult male testes, or anemic adult spleens was digested by *Bam*HI with or without the methylation-sensitive enzyme *Bst*UI. Following size separation in agarose gels, blots were hybridized with α-^32^P-labeled probes and subjected to X-ray autoradiography.

For bisulfite sequencing analysis, genomic DNA from adult male sperm or tail tips was digested with *Xba*I and treated with sodium bisulfite using the EZ DNA Methylation Kit (Zymo Research). Two-cell embryos were embedded in agarose beads and treated with sodium bisulfite as described previously [40]. Subregions of the lambda, LCb or LCb478 fragments were amplified by nested PCR, and the PCR products were subcloned into pGEM-T Easy vector (Promega) for sequence analyses. PCR primers are listed in Tables 1 and 2.

**Table 1:** PCR primer sets for bisulfite sequencing analysis

Regions analyzed	PCR round	5′ primers	3′ primers
I, IV	1st	lambda-MA-5S4	lambda-MA-3A2
	2nd	lambda-MA-5S1	lambda-MA-3A3
II, V	1st	lambda-MA-5S5	lambda-MA-3A7
	2nd	lambda-MA-5S6	lambda-MA-3A8
III, VI, VII	1st	lambda-MA-5S7	BGLB-MA-3A6
	2nd	lambda-MA-5S8	BGLB-MA-3A2

**Table 2:** PCR primer sequences for bisulfite sequencing analysis

	Names	Sequences
5′ primers	lambda-MA-5S1	5′-attagtaagaagatagtagtgatg-3′
	lambda-MA-5S4	5′-ttaagttttgtgtgttatttatta-3′
	lambda-MA-5S5	5′-gttaaaaagaagaagtaagtattt-3′
	lambda-MA-5S6	5′-gtgaaagtattgattattatgtta-3′
	lambda-MA-5S7	5′-gaggtttatttgtatttatttttgtt-3′
	lambda-MA-5S8	5′-tattttttagtagtattgtaagaggt-3′
3′ primers	lambda-MA-3A2	5′-ataccttatttttttctactacaa-3′
	lambda-MA-3A3	5′-ctaaactccaacatataataaccc-3′
	lambda-MA-3A7	5′-aaccaaaattatctttttctatct-3′
	lambda-MA-3A8	5′-acaacattcttaaatccaatatta-3′
	BGLB-MA-3A2	5′-ttctaaccccacaaaaatttattc-3′
	BGLB-MA-3A6	5′-ccaaaccccctctattttatatca-3′

### Chromatin immunoprecipitation (ChIP) assay

The LCb478 YAC-TgM (2–4 months old) inheriting the transgene paternally or maternally were made anemic by phenylhydrazine treatment. Nucleated erythroid cells were collected from their spleens and fixed in PBS with 1% formaldehyde for 10 min at room temperature. Nuclei (2 × 10^7^ cells) were digested with 12.5 units/ml of micrococcal nuclease at 37 °C for 20 min to prepare primarily mono- to di-nucleosome-sized chromatin. The chromatin was incubated with anti-CTCF antibody (D31H2; Cell Signaling Technology) or purified rabbit IgG (Invitrogen) overnight at 4 °C and was precipitated with preblocked Dynabeads protein G magnetic beads (Life Technologies, Carlsbad, CA). Immunoprecipitated materials were then washed extensively and reverse cross-linked. DNA was purified with the QIAquick PCR purification kit (Qiagen, Venlo, the Netherlands) and subjected to qPCR analysis. The endogenous *H19* ICR and *Necdin* sequences were analyzed as positive and negative controls, respectively, [41]. PCR primers were reported previously [41].

### RT-qPCR

Total RNA was recovered from phenylhydrazine-treated anemic adult spleens (1–2 months old) using ISOGEN (Nippon Gene) and converted to cDNA using ReverTra Ace qPCR RT Master Mix with gDNA Remover (TOYOBO). Quantitative amplification of cDNA was performed with the Thermal Cycler Dice (TaKaRa Bio) using SYBR Premix EX TaqII (TaKaRa Bio) and PCR primers listed in Table 3.

**Table 3:** Primer sets for RT-qPCR

Genes	Primer names	Sequences
Human β-*globin*		
5′ primer	BT-1S2	5′-aggagaagtctgccgttactg-3′
3′ primer	BT-1A2	5′-gcccataacagcatcaggagt-3′
Mouse *α*-*globin*		
5′ primer	Hbaa-5S3	5′-agacaaaagcaacatcaagg-3′
3′ primer	Hbaa-3A2	5′-cttggtggtggggaagctag-3′

### EMSA

GST-ZFP57 protein (amino acids 137–195) was expressed in *E. coli* (BL-21/pGEX vector) and purified. Nuclear extracts were prepared from HEK293T cells transfected with a HA-tagged ZFP57 expression plasmid by using Nuclear Extract Kit (Active Motif) according to the manufacturer’s instructions. GST-ZFP57 protein (5 ng) or nuclear extracts (7 µg) were preincubated in the reaction mixture [PBS with 5 mM MgCl_2_, 0.1 mM ZnSO_4_, 1 mM DTT, 0.1% NP40, 10% glycerol, and 1 µg of poly(dI-dC)] for 10 min at RT, with or without 50-fold molar excess of a specific double-stranded competitor DNA. For super-shift assays, 1 µg of anti-HA (12CA5; Roche) or anti-ZFP57 (ab45341; abcam) antibody was included in the reaction mixture. 0.16–1.34 ng (15,000 cpm) of a radiolabeled DNA probe was added and the incubation was continued for 25 min at RT. The incubation mixture was loaded on a 3.5 or 4% non-denaturing polyacrylamide gel in 0.5xTBE buffer, and electrophoresed at 4 °C. The gels were dried and exposed to X-ray film. Probe and competitor sequences are indicated in Fig. 4B.

## Additional files

**Additional file 1: Figure S1.** DNA methylation status of the LCb and LCb478 fragments in somatic cells. (A and C) Partial restriction enzyme maps of the β-globin YAC transgenes with the inserted LCb (A) or LCb478 (C) fragments. Methylation-sensitive *Bst*UI sites in *Bam*HI fragments are displayed as vertical lines beneath each map. (B and D) DNA methylation status of the LCb (B) or LCb478 (D) fragments in tail somatic cells of the YAC–TgM. Tail genomic DNA was digested with *Bam*HI alone (B) or *Bam*HI + *Bst*UI (B + BstUI) and the blots were hybridized with the probe shown in the maps (A and C). Asterisks indicate the positions of parental or methylated, undigested fragments. Individuals inheriting the transgene maternally and paternally are highlighted in pink and blue colors, respectively. In the pedigree, male and female individuals are represented as rectangles and circles, respectively. Filled, gray, or open symbols indicate hyper-, partially, or hypo-methylated status of LCb or LCb478 fragment in each TgM, which was determined by visual examination of the Southern blot results by three individuals. Tail DNA from underlined animals (in the pedigree) was pooled according to the transgene’s parental origin and analyzed by bisulfite sequencing in Fig. 2B, C. Testis samples in Additional file 2: Fig. S2 were obtained from male individuals marked by stars.

**Additional file 2: Figure S2.** DNA methylation status of the LCb and LCb478 fragments in testis. Testis genomic DNA from adult male YAC–TgM was analyzed by Southern blotting as described in the legend to Additional file 1: Fig. S1. Sperm samples were obtained from No. 1578 (LCb, line 469) and 1379 (LCb478, line 469) animals, and methylation status of the transgenes were analyzed by bisulfite sequencing in Fig. 3.

## Abbreviations

ICR: imprinting control region
TgM: transgenic mouse
DMR: differentially methylated region
LCR: locus control region
