# Supplementary figures and images for: Synthetic DNA fragments bearing ICR cis elements become differentially methylated and recapitulate genomic imprinting in transgenic mice
Source: Epigenetics Chromatin. 2018 Jun 29;11:36. doi: 10.1186/s13072-018-0207-z (PMC6027785; doi:10.1186/s13072-018-0207-z)

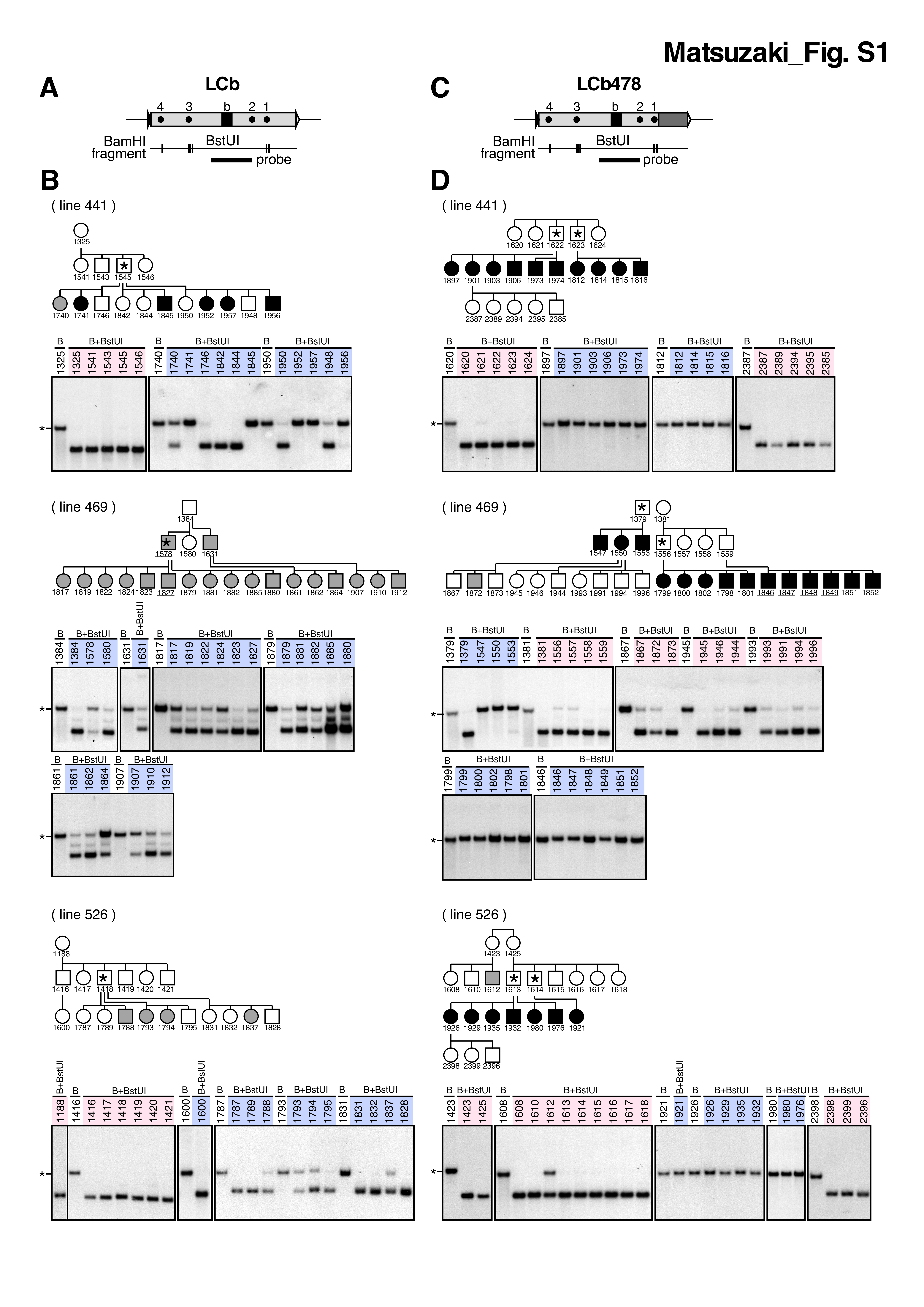

Supplement: Supplementary file 1 — Additional file 1: Figure S1. DNA methylation status of the LCb and LCb478 fragments in somatic cells. (A and C) Partial restriction enzyme maps of the β-globin YAC transgenes with the inserted LCb (A) or LCb478 (C) fragments. Methylation-sensitive BstUI sites in BamHI fragments are displayed as vertical lines beneath each map. (B and D) DNA methylation status of the LCb (B) or LCb478 (D) fragments in tail somatic cells of the YAC–TgM. Tail genomic DNA was digested with BamHI alone (B) or BamHI + BstUI (B + BstUI) and the blots were hybridized with the probe shown in the maps (A and C). Asterisks indicate the positions of parental or methylated, undigested fragments. Individuals inheriting the transgene maternally and paternally are highlighted in pink and blue colors, respectively. In the pedigree, male and female individuals are represented as rectangles and circles, respectively. Filled, gray, or open symbols indicate hyper-, partially, or hypo-methylated status of LCb or LCb478 fragment in each TgM, which was determined by visual examination of the Southern blot results by three individuals. Tail DNA from underlined animals (in the pedigree) was pooled according to the transgene’s parental origin and analyzed by bisulfite sequencing in Fig. 2B, C. Testis samples in Additional file 2: Fig. S2 were obtained from male individuals marked by stars. [file 13072_2018_207_MOESM1_ESM.tif]

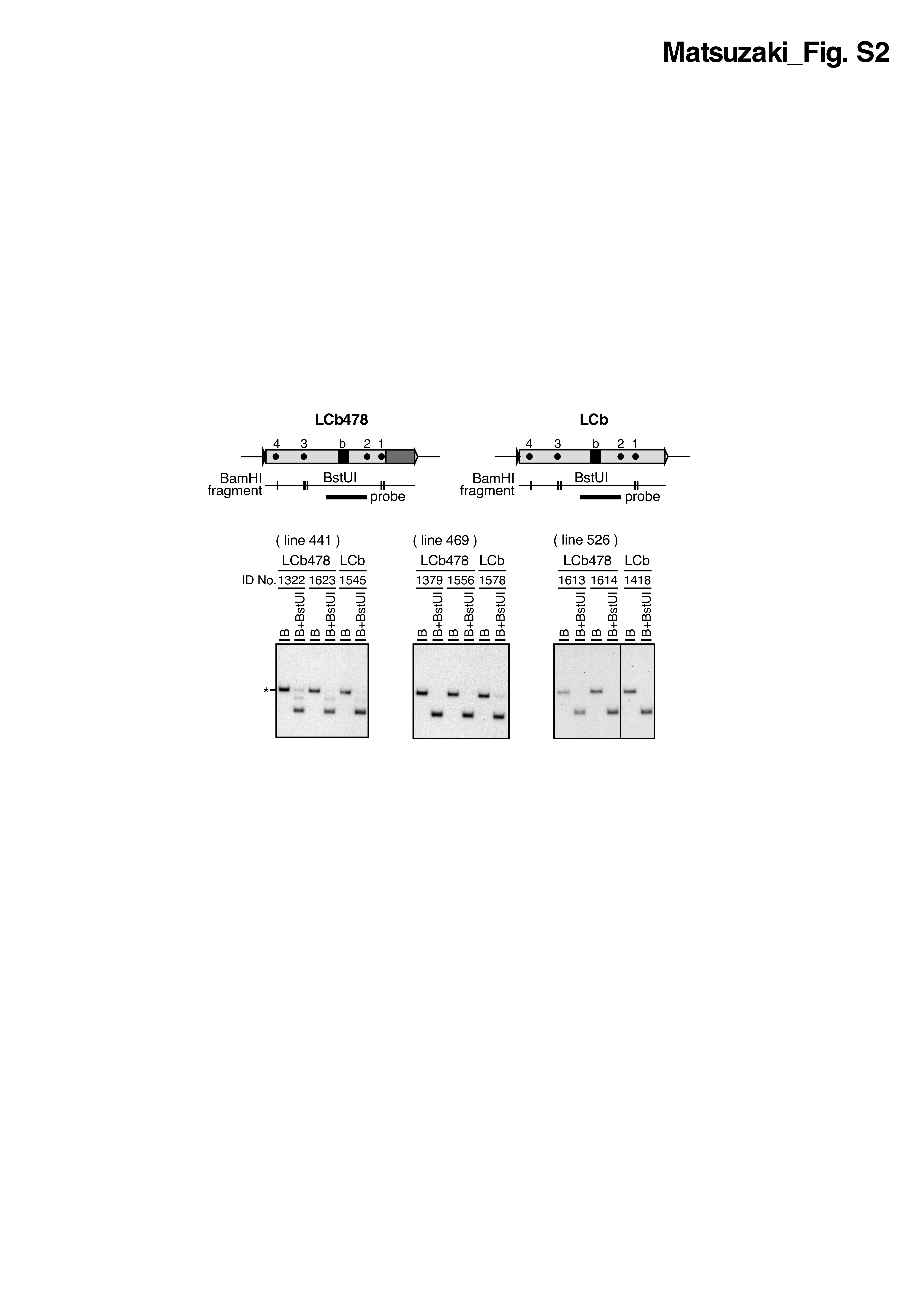

Supplement: Supplementary file 2 — Additional file 2: Figure S2. DNA methylation status of the LCb and LCb478 fragments in testis. Testis genomic DNA from adult male YAC–TgM was analyzed by Southern blotting as described in the legend to Additional file 1: Fig. S1. Sperm samples were obtained from No. 1578 (LCb, line 469) and 1379 (LCb478, line 469) animals, and methylation status of the transgenes were analyzed by bisulfite sequencing in Fig. 3. [file 13072_2018_207_MOESM2_ESM.tif]
